# Melamine Foams Decorated with In-Situ Synthesized Gold and Palladium Nanoparticles

**DOI:** 10.3390/polym12040934

**Published:** 2020-04-17

**Authors:** Javier Pinto, Suset Barroso-Solares, Davide Magrì, Francisco Palazon, Simone Lauciello, Athanassia Athanassiou, Despina Fragouli

**Affiliations:** 1 Smart Materials, Istituto Italiano di Tecnologia, Via Morego 30, 16163 Genova, Italy; sbarroso@fmc.uva.es (S.B.-S.); davide.magri@ec.europa.eu (D.M.); simone.lauciello@iit.it (S.L.); Athanassia.Athanassiou@iit.it (A.A.); 2 Cellular Materials Laboratory (CellMat), Condensed Matter Physics Department, University of Valladolid, 47011 Valladolid, Spain; 3 Nanochemistry Department, Istituto Italiano di Tecnologia, Via Morego 30, 16163 Genova, Italy; francisco.palazon@uv.es; 4 Instituto de Ciencia Molecular, Universidad de Valencia, C/Beltrán 2, 46980 Paterna, Spain

**Keywords:** noble metal nanoparticles, polymer foam, polydimethylsiloxane, in-situ

## Abstract

A versatile and straightforward route to produce polymer foams with functional surface through their decoration with gold and palladium nanoparticles is proposed. Melamine foams, used as polymeric porous substrates, are first covered with a uniform coating of polydimethylsiloxane, thin enough to assure the preservation of their original porous structure. The polydimethylsiloxane layer allows the facile in-situ formation of metallic Au and Pd nanoparticles with sizes of tens of nanometers directly on the surface of the struts of the foam by the direct immersion of the foams into gold or palladium precursor solutions. The effect of the gold and palladium precursor concentration, as well as the reaction time with the foams, to the amount and sizes of the nanoparticles synthesized on the foams, was studied and the ideal conditions for an optimized functionalization were defined. Gold and palladium contents of about 1 wt.% were achieved, while the nanoparticles were proven to be stably adhered to the foam, avoiding potential risks related to their accidental release.

## 1. Introduction

Noble metal nanoparticles (NPs) have attracted considerable attention in the last years due to their unique physical properties and potential for diverse novel applications [1,2,3,4,5,6,7,8,9]. These NPs present not only a high surface to volume ratio but also different properties than bulk materials, such as surface plasmon resonance, surface-enhanced Raman scattering, variations in the band gap, while keeping high stability [1,2,3,4]. Therefore, noble metal NPs have proved to be suitable materials for biomedical [1,6,10,11], sensoring [3,8,12,13,14], optoelectronics [15,16], water remediation [5,17,18,19], catalysis [7,20,21,22], and food control applications [4]. 

However, the direct use of free-standing NPs raises some concerns, mainly related to their efficiency, safety, and recovery [15,23,24]. On the one hand, free-standing NPs usually tend to aggregate, a fact that causes the decrease of their active surface area and the modification of their key physical properties (e.g., photocatalysis), therefore, their performance in some applications (e.g., water remediation) is compromised [15,23,24]. On the other hand, after their use on water treatment, sensoring, or catalysis procedures, time-consuming and complicated actions need to be adopted for their recovery, as their accidental release to the environment could be toxic for several organisms [25].

The development of polymeric nanocomposites has been identified as a promising approach to overcome these drawbacks. Incorporating the noble metal NPs into a polymer matrix allows anchoring and stabilizing the NPs, avoiding their aggregation or accidental release, and facilitates their quick recovery [5,10,20,23,26,27,28,29,30]. However, a proper nanocomposite fabrication route is a key factor for the maintenance of the NPs functionality. Traditional polymer nanocomposite fabrication routes, such as mechanical blending processes (e.g., extrusion) or mixing in solution followed by casting, present several disadvantages. First, an optimum NPs dispersion into the polymer is difficult to achieve, and the NPs usually form aggregates unless specific functionalization procedures are adopted for the enhancement of their compatibility with the polymer matrix [29,31,32]. Second, most of the NPs incorporated in the composites by these procedures, are imbibed into the polymer, hindering their direct interaction with the environment, which is required for several applications such as sensoring, catalysis, and water treatment [5,29]. Accordingly, functionalization approaches aiming to anchor or create the NPs directly onto the surface of polymeric substrates are necessary to develop efficient nanocomposites for these applications [5,10].

Among other polymeric substrates, open-cell polymer foams offer an interconnected porous structure with a high surface area for the anchoring of NPs [5], making them excellent substrates to develop highly efficient polymer nanocomposites for diverse applications. For instance, polymer foams decorated with Ag NPs with water disinfection capability have been produced using dip-coating and in-situ NPs synthesis approaches [5,23,26,27,28]. The best results, in terms of both high anchoring stability and amount of Ag NPs on the surface of the foams, were obtained by the in-situ synthesis of the Ag NPs directly on the surface of the polymer foams struts [5,23]. However, the functionalization of polymer foams with other noble metal NPs, such as Au or Pd, and therefore their potential use in different applications, is less studied. In the case of Au NPs, Apyari et al. [33] immersed polyurethane foams in dispersions of Au NPs, promoting their adsorption into the surfaces of the foam and using them as an optical sensor of organic compounds. Calcagnile et al. [34] produced polydimethylsiloxane (PDMS) sponges using hydrogel calcium alginate beads as a template for the porous structure. The incorporation of HAuCl_4_ into the beads allowed the in-situ formation of the Au NPs onto the surface of the cells of the PDMS sponges. Gupta and Kulkarni [35] followed a similar approach, adding the Au precursor directly into the PDMS formulation, obtaining nanocomposite sponges able to remove some organic compounds from polluted water. Concerning the Pd NPs, Desforges et al. [36] functionalized microcellular Poly(styrene/DVB) with Pd NPs formed in-situ directly on the foams’ surface upon their immersion into a K_2_PdCl_4_ solution, being the Pd NPs synthesis spontaneously carried on upon 15 days of interaction or accelerated by adding NaBH_4_ or using UV light. These materials were employed as catalyzers in hydrogenation reactions. Additionally, Suhaimi et al. [37] produced nanocomposite polysulfone membranes for gas separation by adding a Pd precursor to the polymer formulation prior to the fabrication of the membranes. Among these works, only Apyari et al. [33] and Desforges et al. [36] proposed functionalization procedures that do not require the modification of the production route of the porous substrates. Moreover, the approach proposed by Apyari et al. [33] employed commercially available polymer foams, facilitating, thus, the scale-up of the developed materials and their wide applicability.

Herein, a facile two-step route to decorate polymer foams with Au and Pd NPs is presented, using commercially available melamine (ME) foams as the porous substrate. The proposed procedure takes advantage of the chemical reduction of Au and Pd precursors induced by the functional groups of a thin polydimethylsiloxane coating [10,30,34], that was previously performed on the struts of the ME foams. The proposed procedure allows synthesizing a large number of Au or Pd NPs, with average particle sizes below 50 nm, on the foams’ surface, and without affecting their porous structure. Moreover, as proved, the noble metal NPs are exposed in the surface of the struts and well anchored to the foam, without any accidental NPs release even after prolonged treatments with water. Therefore, the proposed functionalization procedure overcomes the conventional drawbacks of the production of polymer nanocomposites, providing noble metal nanocomposite ME-based foams with optimal features for water treatment, sensoring, and catalysis.

## 2. Materials and Methods 

### 2.1. Materials 

Commercial grade Basotec G+ (BASF, Schwarzheide, Germany) Melamine (ME) foams with very high porosity (0.994), open cellular structure, and a broad cell size distribution (average cell bellow 500 µm) were kindly provided by LAPE HD Srl (Empoli, Italy). PDMS Sylgard 184 kit, composed by a PDMS base and a curing agent, was purchased from Dow Corning (Midland, MI, US). Chloroauric acid (HAuCl_4_) and sodium tetrachloropalladate (Na_2_PdCl_4_) were purchased from Sigma-Aldrich (St. Louis, MO, US) and used as Au and Pd NPs precursors, respectively. Ethyl acetate and ethanol were also purchased from Sigma-Aldrich (St. Louis, MO, US) and used as solvents. Distilled water was employed for the rinsing of the foams.

### 2.2. Functionalization of the ME Foams 

Samples (2 × 1 × 1 cm^3^) of the pristine ME foams were rinsed in ethanol, dried, and then immersed for 3 min into ethyl acetate solutions containing 1 v.% of the PDMS base and the curing agent in a weight ratio 10:1. Subsequently, the samples were extracted from the solution and cured for 3 h at 80 °C (these processing parameters were previously optimized, see Supplementary Information, Figure S1). Then, the obtained ME/PDMS foams were rinsed with ethanol to remove any unreacted PDMS. The in-situ synthesis of Au or Pd NPs on the ME/PDMS foams (2 × 1 × 1 cm^3^) was achieved by immersing them on 20 mL ethanol solutions of HAuCl_4_ or Na_2_PdCl_4_, respectively. Four different concentrations of each precursor were employed: 0.175, 0.350, 0.700, and 1.400 mg/mL of HAuCl_4_ and 0.250, 0.500, 1.000, and 2.000 mg/mL of Na_2_PdCl_4_. These concentrations were determined to ensure a comparable availability of Au and Pd ions in the solutions. The immersed ME/PDMS samples were kept under shaking for different reaction times (ranging from 1 to 120 h). Then, the samples were extracted and subjected to five washing cycles. In each washing cycle, the samples were immersed in 20 mL of distilled water for 2 min under shaking. This washing procedure was required to ensure the complete removal of ethanol or NPs unreacted precursor from the foams, as well as of non-well attached NPs (see Supplementary Information, Figure S2).

### 2.3. Experimental Techniques

The porous structure of the ME foams, the surface and thickness of their struts, and the presence and size of Au and Pd NPs were studied by High-Resolution Scanning Electron Microscopy (HRSEM) using a JEOL JSM-7500La (Jeol, Tokyo, Japan) equipped with a cold field-emission gun (FEG), operating at 15 kV acceleration voltage. The thickness of the struts was measured at the intermediate point between vertices. The thickness distribution of the struts was determined from several micrographs of each foam, by analyzing at least 100 struts in each case using FIJI/ImageJ. [38] Micrographs were obtained using backscattered electrons, whereas energy-dispersive spectroscopy (EDS) was employed to study the distribution of the Au and Pd elements on the struts of the foams. The size distribution, average size, and standard deviation of the formed Au and Pd NPs for each preparation conditions were determined from several micrographs for each foam, by analyzing at least 100 NPs in each case using FIJI/ImageJ. [38] The PDMS content of the ME/PDMS foams was determined by weighing the samples before and after the formation of the PDMS coating. Moreover, the Au and Pd NPs content of the ME/PDMS/Au and ME/PDMS/Pd foams were evaluated using Inductive Couple Plasma-Optical Emission Spectroscopy (ICP-OES, iCAP 3600 spectrometer, Thermo Fisher Scientific, Waltham, MA, USA). A microwave digestion system (MARS Xpress, CEM, Matthews, NC, US) was employed to digest 5 mg of foams in 2.5 mL of nitric acid (70%, Sigma Aldrich, St. Louis, MO, US). The solid degradation reaction was performed at 180 °C for 15 min. Then, the samples were diluted in Milli-Q water up to 25 mL and filtered through polytetrafluoroethylene (PTFE) syringe filters (15 mm, pore size 0.45 µm, Sartorius, Göttingen, Germany). The surface chemistry of the ME/PDMS/Au and ME/PDMS/Pd foams was studied by X-ray Photoelectron Spectroscopy (XPS) using an Axis Ultra DLD spectrometer from Kratos® (Manchester, UK) under 10^−9^ mbar pressure and equipped with a monochromatic Al Kα source (photon energy = 1486.6 eV, emission current = 20 mA, and operation voltage = 15 kV). High-resolution spectra of these materials were obtained with a step of 0.1 eV and an analyzer pass energy of 10 eV. Surface charging was neutralized with low-energy electrons (4 eV), and the energy calibration was carried out by setting the C-C/C-H component of the C 1 s spectrum to a fixed binding energy value of 284.5 eV. Data analysis was performed with CasaXPS software.

## 3. Results and Discussion

### 3.1. ME/PDMS Foams

**Figure 1:**
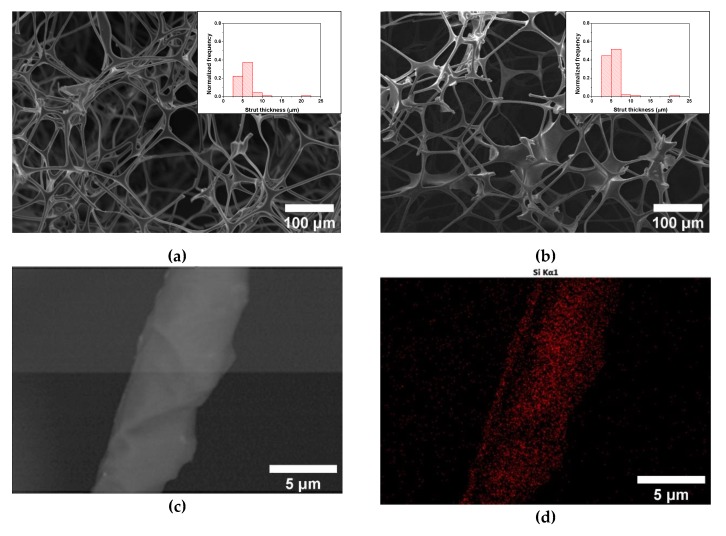

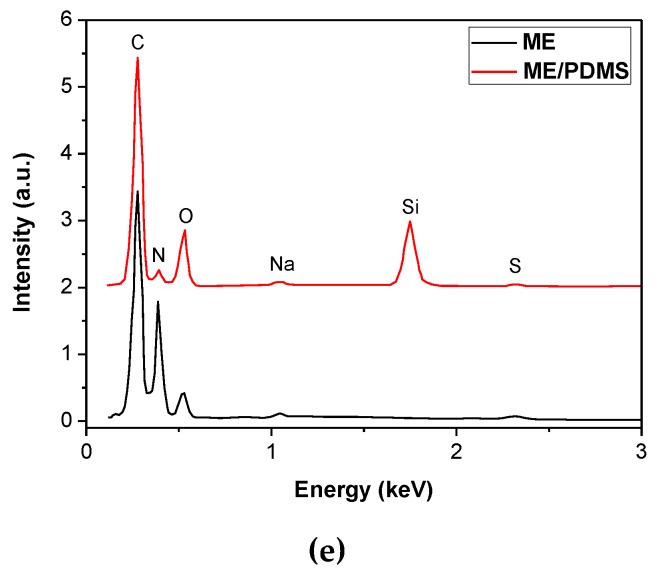
High-resolution Scanning Electron Microscopy (HRSEM) micrographs of the untreated Melamine (ME) (**a**) and ME/Polydimethylsiloxane (PDMS) cured at 80 °C (**b**). HRSEM image of a strut of the ME/PDMS foam (**c**) and the corresponding energy-dispersive spectroscopy (EDS) map (**d**) showing a homogeneous distribution of the Si corresponding to the PDMS (red). EDS spectra of ME and ME/PDMS foams (**e**).

The efficiency of the proposed procedure to create a PDMS coating over the struts of the ME foams was studied. First, it was determined that the amount of PDMS transferred to the foam was 104 ± 13 wt.%, from which a stable PDMS coating of about 97 ± 13 wt.% remained after the ethanol rinsing. As previously proved in other polymer foams, the PDMS layer is expected to establish H-bonds with the polymer foam substrate [39]. This amount of PDMS was found to be enough to provide a thin homogeneous layer covering the struts of the ME foams, without inducing a significant thickness increase or clogging of the porous structure (Figure 1a,b). From the morphological analysis of the foams, the PDMS layer is expected to have a thickness below a micron, as no significant difference was found in terms of the struts thicknesses distribution (Figure 1a,b) or they average size (average values of 5.8 ± 2.3 and 5.3 ± 1.8 µm respectively, for ME and ME/PDMS foams). The EDS analysis of the surface of the struts shows that the Si signal, representative of the PDMS coating, is homogeneously distributed along the struts of the ME/PDMS foams (Figure 1c,d). From the corresponding EDS spectra of the foams, clear differences are shown, as in the case of ME/PDMS foams, the Si signal is present together with a significantly lower signal of the N of the ME substrate (Figure 1e). Therefore, with this step, a stable PDMS layer homogeneously distributed on the ME foams is successfully formed, preserving the high surface area and porosity of the ME and providing a platform for the in-situ synthesis of noble metal nanoparticles. 

### 3.2. ME/PDMS/Au and ME/PDMS/Pd Foams

**Figure 2:**
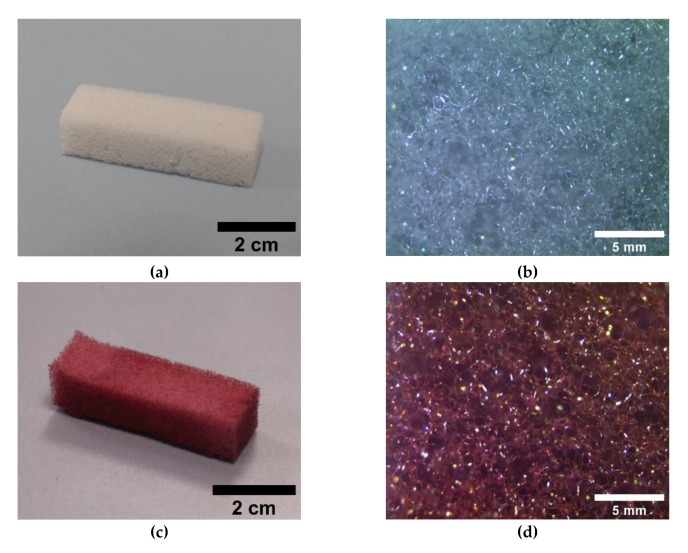

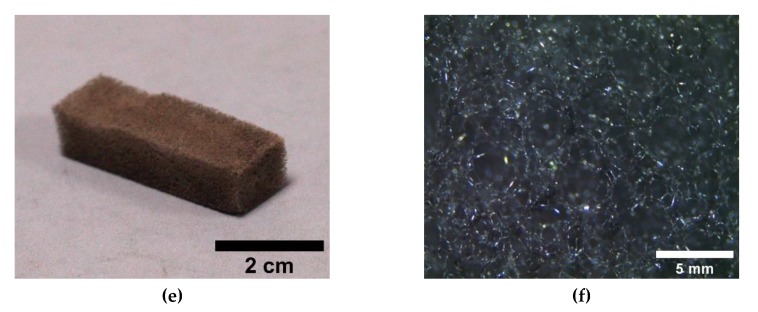
Photographs of the untreated ME (**a**) and treated ME/PDMS/Au (**c**) and ME/PDMS/Pd (**e**) foams. Optical micrographs (40×) of the untreated ME (**b**) and treated ME/PDMS/Au (**d**) and ME/PDMS/Pd (**f**) foams showing the homogenous color of the struts of the foams.

The treatment of ME/PDMS foams with the HAuCl_4_ solutions induced an evident change in the color of the foams, from the light gray of ME/PDMS (Figure 2a,b) to red (Figure 2c,d), a clear indication of the presence of Au NPs [33,34,35]. As shown by the photographs (Figure 2c) and the optical micrographs (Figure 2d), the coloration of the foams is homogeneous, which is an indication that the expected presence of Au NPs is throughout the porous structure of the foams. On the other hand, the treatment of the ME/PDMS foams with Na_2_PdCl_4_ solutions also induced a color change, with the ME/PDMS/Pd foams showing a uniform dark grey-blackish color (Figure 2e,f), related to the presence of Pd NPs [36]. 

No significant weight changes were found during the production of the ME/PDMS/Au and the ME/PDMS/Pd foams from the ME/PDMS foams, so moderate Au or Pd loads are expected on these samples. This amount of Au or Pd transferred to the foams, upon dipping to the precursor solutions of different concentrations and for different time intervals, was accurately determined by the ICP-OES analysis of the ME/PDMS/Au and ME/PDMS/Pd foams. After 48 h of immersion of the foams in solutions with HAuCl_4_ concentrations ranging from 0.175 to 1.400 mg/mL, the transferred Au to the foams was ranging from c.a. 0.6 to 1.0 wt.%. For the precursor concentration of 0.350 mg/mL, the Au transfer already reached c.a. 1 wt.%, and this concentration was chosen for the further analysis of the kinetics of the Au transfer onto the foams. Similarly, ME/PDMS/Pd foams obtained after 48 h of immersion in solutions with Na_2_PdCl_4_ concentrations ranging from 0.250 to 2.000 mg/mL showed Pd contents ranging from 0.8 to 1.2 wt.%. Additionally, an intermediate concentration of 0.500 mg/mL, which provides a Pd transfer c.a. 1.1 wt.%, was chosen to study the kinetics of the procedure. It should be noticed that this concentration is also equivalent to the selected Au concentration (i.e., 0.350 mg/mL) in terms of the noble metal ions availability, about 0.200 mg/mL. Thus, a direct comparison between the ME/PDMS/Au and ME/PDMS/Pd foams can be performed. 

**Figure 3:**
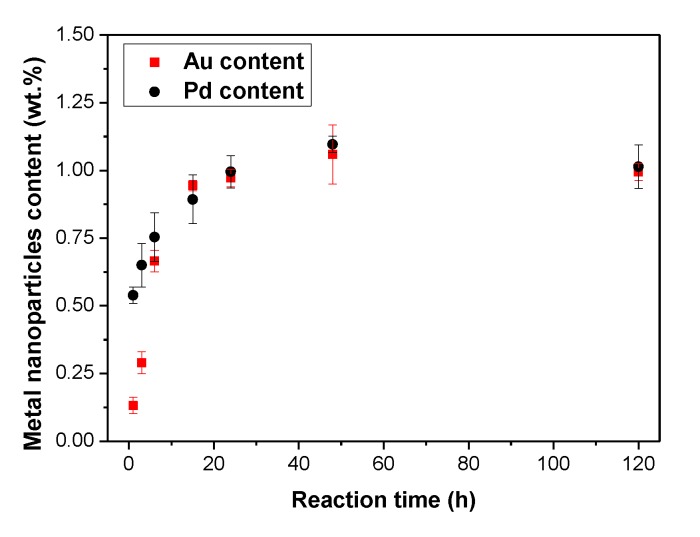
Au and Pd content (wt.%) measured by Inductive Couple Plasma-Optical Emission Spectroscopy (ICP-OES) in the treated ME/PDMS/Au and ME/PDMS/Pd foams obtained after different immersion times in 0.35 mg/mL of HAuCl_4_ and 0.50 mg/mL of Na_2_PdCl_4_ in ethanol, respectively.

In particular, ME/PDMS/Au and ME/PDMS/Pd foams were produced upon the dipping of the ME/PDMS foams in the HAuCl_4_ or Na_2_PdCl_4_ solutions for time intervals ranging from 1 to 120 h. As shown in Figure 3, both the Au and Pd amounts transferred to the foam increased with the reaction time increase, starting from about 0.1 wt.% Au and 0.5 wt.% Pd after 1 h of reaction time and reaching a stable value of about 1 wt.% Au and 1.1 wt.% Pd after 48 h. According to these values, the Pd transfer seems to be faster in the beginning, reaching almost 50% of the final load in just 1 h, while the Au transfer reaches about 10% at the same time. However, after 15 h, the transferred amounts of both noble metals became similar.

**Figure 4:**
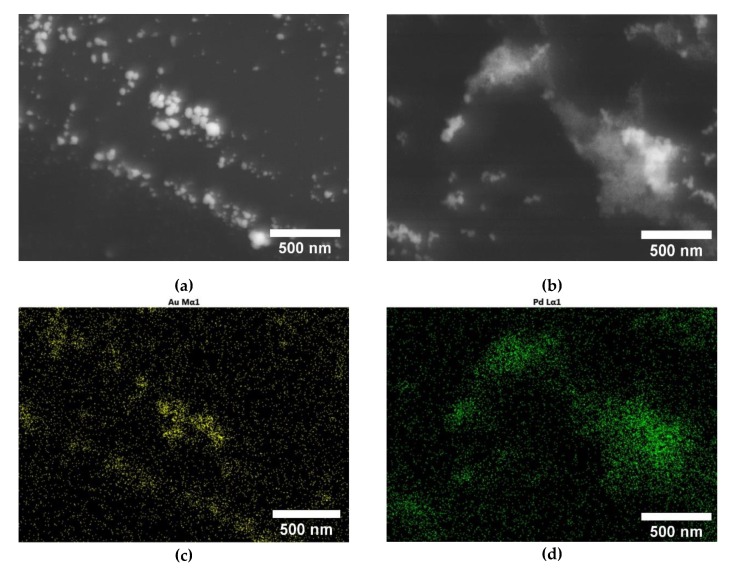
HRSEM micrographs of the struts of the ME/PDMS/Au (**a**) and ME/PDMS/Pd foams (**b**) showing the presence of small nanoparticles (NPs) and aggregates. EDS maps of the micrographs showing of the ME/PDMS/Au **(c)** and ME/PDMS/Pd (**d**) showing the Au (**c**) or Pd (**d**) signal of the NPs.

Previous studies have shown that the functional components of the PDMS are inducing the formation of noble metal NPs on their surface upon chemical reduction of the metallic precursors adsorbed. In particular, the noble metal nanoparticles formation is expected to happen in non-reacted cross-linking Si-H sites [10,30,34,35]. In order to prove, in the present case, the transformation of the adsorbed Au or Pd ions into NPs onto the surface of the foams in the presence of PDMS, detailed HRSEM and EDS studies were performed. As shown in Figure 4, both ME/PDMS/Au and ME/PDMS/Pd foams present small NPs and aggregates on the surface of their struts. Moreover, EDS mapping of the struts of the ME/PDMS/Au and ME/PDMS/Pd foams confirmed that both the small NPs and bigger aggregates are attributed to the presence of Au or Pd NPs (Figure 4c,d, respectively). The presence of Au or Pd NPs in their crystalline form was further confirmed by X-ray Diffraction analysis in all cases (XRD, see Supplementary Information, Figure S3). It should be mentioned that the presence of Au or Pd NPs is strictly related to the PDMS layer, as the EDS elemental maps obtained by HRSEM analysis of ME and ME/PDMS foams proved. In fact, the NPs are present only on the latter foams (see Supplementary Information, Figure S4). In contrast, pure ME foams subjected to the same dipping procedure presented no modifications on their surface.

**Figure 5:**
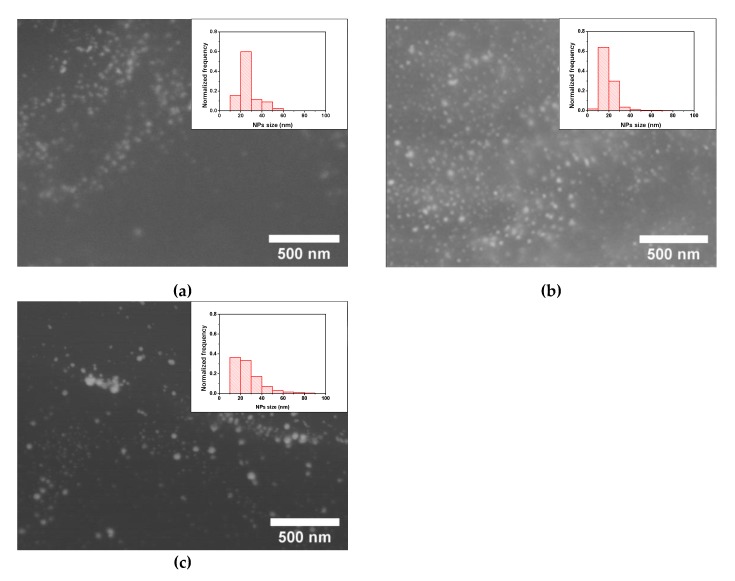
HRSEM micrographs and NPs size distribution (insets) of the ME/PDMS/Au foams obtained after different reaction times in 0.35 mg/mL of HAuCl_4_ in ethanol: 1 (**a**), 24 (**b**), and 120 h (**c**).

Regarding the effect of the reaction time for a fixed concentration of 0.350 mg/mL of HAuCl_4_, quite constant average sizes between 18 and 20 nm and similar size distributions with most of the NPs ranging from 10 to 30 nm were found for reaction times between 1 and 48 h (Figure 5 and Figure S6, see Supplementary Information). This result indicates that the significant increase of Au transferred to the foams occurring in that range, from 0.1 to 1 wt.% (Figure 3), is related to the rise of the number of Au NPs synthesized and not to their size increase. Longer reaction times provided a slightly larger average size of about 25 nm and a broadening of the size distribution with a noticeable rise in the presence of NPs over 30 nm (Figure 5). Therefore, taking into account the results of the amount of Au transferred and the NPs sizes the optimal conditions to produce the ME/PDMS/Au foams are a HAuCl_4_ concentration of 0.350 mg/mL and 48 h of reaction time, obtaining 1 wt.% of Au NPs with sizes about 18 nm synthesized on the foams. The achieved amount of Au NPs synthesized on the foams is comparable to the results obtained by Apyari et al. [33], about 0.9 wt.%, and significantly higher than those of Gupta and Kulkarni [35] (0.06 wt.%). 

**Figure 6:**
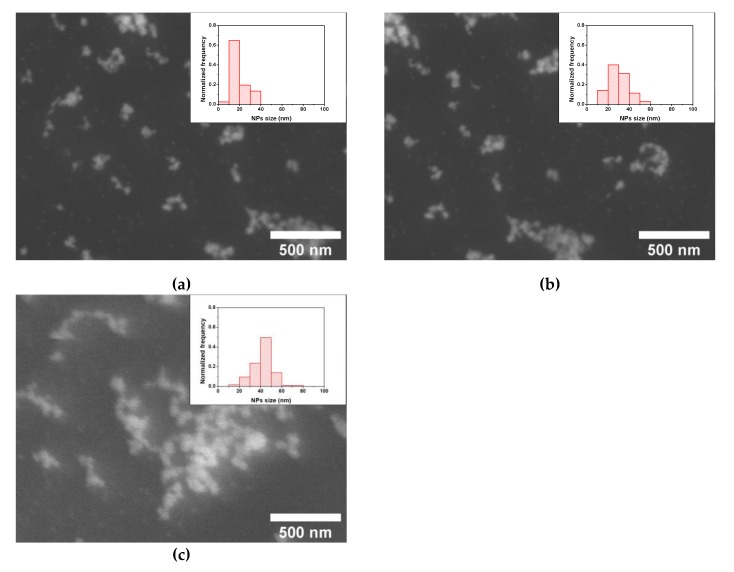
HRSEM micrographs and NPs size distribution (insets) of the obtained ME/PDMS/Pd foams obtained after different reaction times in 0.50 mg/mL of Na_2_PdCl_4_ in ethanol: 1 (**a**), 24 (**b**), and 120 h (**c**).

On the contrary, the reaction time between the ME/PDMS foams and the 0.500 mg/mL Pd precursor solutions present a stronger influence on the Pd NPs size than in the case of Au NPs. Reaction times from 1 to 24 h provided NPs average sizes about 27 to 30 nm (Figure 6 and Figure S7, see Supplementary Information), while longer times (48 and 120 h) produced Pd NPs with average sizes about 42 to 45 nm (Figure 6 and Figure S7, see Supplementary Information). In all the cases, the NPs size distributions present similar widths, with no clear relationship with the reaction time. Accordingly, in this case, it is possible to obtain ME/PDMS/Pd foams with similar Pd loads of about 1.0 to 1.1 wt.% (Figure 3) and different particle sizes, about 30 or 45 nm, by using reaction times of 24 or 48 h, respectively. The obtained results, in terms of the amount of Pd transferred to the foams, are lower than previous results reported in the literature using porous polymeric substrates (6.7 wt.% using Poly(styrene/DVB)/PolyHIPE and 3 wt.% using Polysulfone membranes) [36,37]. However, these previous approaches cannot be applied to commercially available substrates or ensure the presence of the Pd NPs on the surface of the struts of the porous substrates.

In addition, the proposed in-situ synthesis procedure ensures not only the presence of the Au or Pd NPs on the surface of the struts of the foams but also their stable anchoring, as the obtained nanocomposite foams do not release the NPs even if they are subjected to shaking in water during 24 h (see Supplementary Information, Figures S2 and S6). Therefore, these foams could be safely employed with any aim without risks of accidental release of the NPs to the environment.

### 3.3. XPS Study of the ME/PDMS/Au and ME/PDMS/Pd Foams

**Figure 7:**
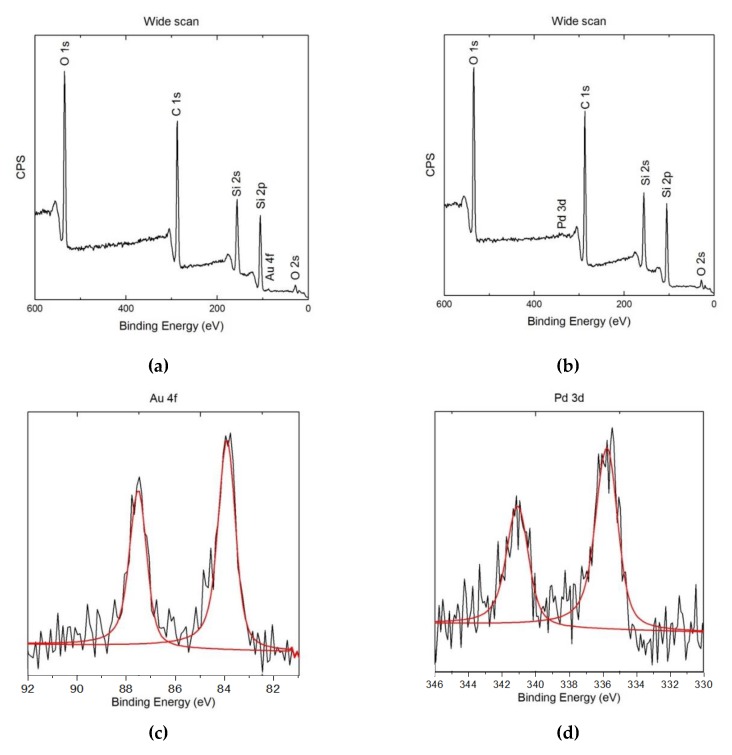
X-ray Photoelectron Spectroscopy (XPS) wide spectra of ME/PDMS/Au (**a**) and ME/PDMS/Pd (**b**) foams. High-resolution Au 4f (**c**) and Pd 3d peaks (**d**) and their deconvolution.

The surface of the obtained ME/PDMS/Au and ME/PDMS/Pd foams was further analyzed by XPS (Figure 7). After the treatment, the wide scan spectra confirm the presence of PDMS and of the metals on the surface of their struts for both foams. In fact, as shown in Figure 7a,b, the Si 2s and Si 2p peaks appear in both cases as well as the Au 4f and Pd 3d peaks for each type of foam. High-resolution XPS spectra of the Au and Pd peaks was performed to determine whether the Au and Pd signals come from metallic NPs or unreacted residual precursors (Figure 7c,d). On the one hand, the Au 4f spectrum of the ME/PDMS/Au foams was accurately fitted with the peaks corresponding to 4f_7/2_ and 4f_5/2_ of metallic gold (Au^0^), with binding energies of 84.0 and 87.7 eV, proving the metallic character of the obtained Au NPs [40]. No residues from the Au precursor (i.e., Cl signal) were found on the foams (see Supplementary Information, Figure S8). On the other hand, the characteristic Pd 3d_5/2_ and Pd 3d_3/2_ peaks of metallic Pd particles appeared respectively about 335.5–335.9 and 340.8–341.2 eV. The obtained values seem to be slightly shifted from the position of metallic Pd (335.2 and 340.5 eV) [36]. This shift of the energies of the peaks could be related to the presence of not only metallic Pd, but also a small amount of some Pd compound. The potential presence of residues of the Pd precursor, Na_2_PdCl_4_, can be discarded as no Cl residues from the Pd precursor were found on the foams by XPS analysis (see Supplementary Information, Figure S8). On the contrary, according to previous works, this effect can be related to the spontaneous formation of a thin oxide layer (PdO, with 3d_5/2_ and 3d_3/2_ energies of 336.8 and 342.1 eV) around the Pd NPs [36]. Although this outer oxide layer could negatively affect the performance of the Pd NPs on different applications, it has been previously reported that the NPs are still active on catalysis procedures [36].

Therefore, the proposed approach proved to be a suitable route to obtain nanocomposite polymer foams with a large number of noble metal NPs (up to 10^14^ particles/m^2^, according to SEM observations (Figure 5 and Figure 6)), specifically located and stably anchored on the struts of the foams. Additionally, this versatile route can be applied to commercially available porous substrates other than ME foams (see Supplementary Information, Figure S9), without modifying their porous structure, proving the applicability and scalability of this approach, as well as the possibility to be used in diverse applications such as water treatment, catalysis, or sensoring.

## 4. Conclusions

This work proposed a facile two-step route to produce polymer foams decorated with Au or Pd nanoparticles (ME/PDMS/Au or ME/PDMS/Pd foams). The metallic nanoparticles were formed in-situ onto the surface of the struts of the foams, which in a previous step were covered by a thin layer of PDMS. The presence of Au and Pd nanoparticles, with mean sizes respectively of about 18 and 30 to 45 nm, over this PDMS layer, was confirmed by electron microscopy studies. In addition, the metallic character of the noble metal nanoparticles was demonstrated by XRD and XPS, although the Pd nanoparticles seem to show a thin oxidized outer layer. It was proved that 0.350 mg/mL and 0.500 mg/mL solutions, respectively of the Au and Pd precursors, provide enough availability of metal ions for the formation of the nanoparticles, making it possible to control the amount of metal nanoparticles synthesized by adjusting the reaction times between 1 and 48 h. Maximum Au and Pd loads reached on the ME/PDMS/Au and ME/PDMS/Pd foams were about 1 and 1.2 wt.%, respectively.

Moreover, the adhesion of the Au and Pd nanoparticles to the foams was proved to be stable. Thus, the proposed approach is a promising and versatile route to produce nanocomposite polymer foams with metallic Au or Pd nanoparticles specifically located on the outer surfaces of the struts of the foams. Therefore, the obtained nanocomposite foams can be suitable and safe materials on several applications on which Au and Pd have shown remarkable performances, such as water treatment, catalysis, and sensoring.

## Supplementary Materials

The following are available online at https://www.mdpi.com/2073-4360/12/4/934/s1, additional details about the ME/PDMS, ME/PDMS/Au, and ME/PDMS/Pd foams fabrication. XRD characterization of the ME/PDMS/Au and ME/PDMS/Pd foams. Additional details of the XPS characterization of ME/PDMS/Au and ME/PDMS/Pd foams. Additional information about the use of the proposed approach in polyurethane foams. Figure S1: HRSEM micrograph of a ME/PDMS foam obtained with PDMS contents in ethyl acetate over 1 v.% showing significant clogging of the porous structure, Figure S2: UV-Vis spectra of the remaining water after 1, 2, 3, 4, and 5 rinsing cycles with distilled water of a ME/PDMS/Au foam (about 1 gold wt.%). As observed, after five rinsing cycles, no signal from residues or NPs can be detected (no relevant signals are present over 250 nm). On the contrary, from one to four rinsing cycles, a peak can be observed about 530 nm, which is related to non-attached gold NPs. Additionally, once the rinsing procedure is completed, the samples can be kept in water under shaking for 24 hours without releasing NPs or any residues, Figure S3: X-ray diffractograms of the ME/PDMS/Au **(a)** and ME/PDMS/Pd **(b)** foams, showing the characteristic peaks of metallic Au and Pd, Figure S4: HRSEM micrograph of a ME/PDMS/Au foam obtained after 48 hours in contact with a 0.70 mg/mL solution of HAuCl_4_ in ethanol **(a)** and the corresponding EDS maps showing the direct relationship between the presence of Si from the PDMS **(b)** and the formation of Au NPs **(c)**. SEM micrograph of a ME/PDMS/Pd foam obtained after 15 hours in contact with a 0.50 mg/mL solution of Na_2_PdCl_4_ in ethanol **(d)** and the corresponding EDS maps showing the direct relationship between the presence of Si **(e)** from the PDMS and the formation of Pd NPs **(f)**, Figure S5: HRSEM micrograph of the struts of a ME/PDMS/Au foam before **(a)** and after **(b)** being kept in water for 24 under shaking, showing no alteration of the presence of NPs on the struts, Figure S6: HRSEM micrographs and NPs size distribution (insets) of the ME/PDMS/Au foams obtained after different reaction times in 0.35 mg/mL of HAuCl_4_ in ethanol: 3 **(a)**, 6 **(b)**, 15 **(c)**, and 48 h **(d),** Figure S7: HRSEM micrographs and NPs size distribution (insets) of the obtained ME/PDMS/Pd foams obtained after different reaction times in 0.50 mg/mL of Na_2_PdCl_4_ in ethanol: 3 **(a)**, 6 **(b)**, 15 **(c)**, and 48 h **(d),** Figure S8: High-resolution XPS spectra of ME/PDMS/Au **(a)** and ME/PDMS/Pd **(b)** centered at binding energies corresponding to Cl 2p showing no remaining of the precursor salts, Figure S9: HRSEM micrographs of the surface of the struts of PU/PDMS/Au **(a)** and PU/PDMS/Pd **(b)** foams showing the presence of Au and Pd NPs, respectively.
