# Supplementary material for: Melamine Foams Decorated with In-Situ Synthesized Gold and Palladium Nanoparticles
Source: Polymers (Basel). 2020 Apr 17;12(4):934. doi: 10.3390/polym12040934 (PMC7240623; doi:10.3390/polym12040934)
Supplement: Supplementary file 1 [file polymers-12-00934-s001.pdf]

polymers

Supplementary Information

Melamine Foams Decorated with *in-Situ* Synthesized Gold and Palladium Nanoparticles

Javier Pinto*, Suset Barroso-Solares, Davide Magrì, Francisco Palazon, Simone Lauciello, Athanassia Athanassiou, and Despina Fragouli*.

Preparation of ME/PDMS foams

In a preliminary stage, it was found that 1 v.% is the optimal amount of PDMS in the ethyl acetate solution to cover the struts of the ME foams without inducing a significant clogging of the porous structure (Figure S1).

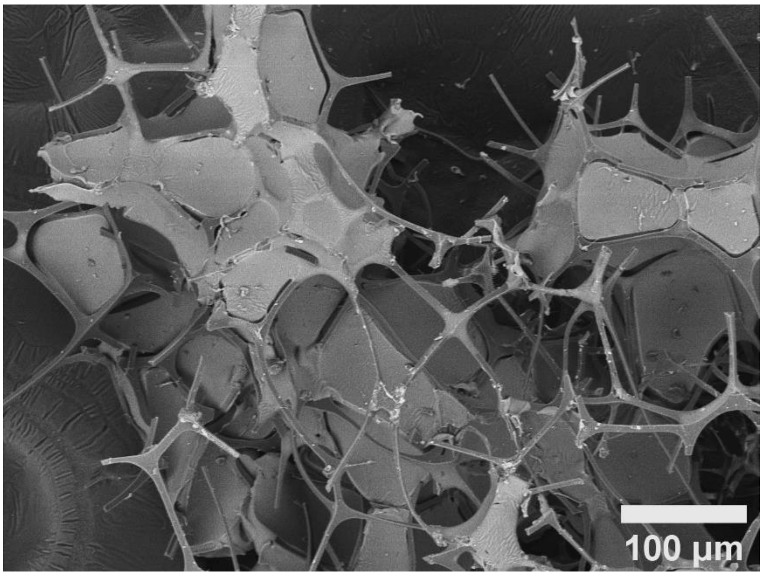

Figure S1. HRSEM micrograph of a ME/PDMS foam obtained with PDMS contents in ethyl acetate over 1 v.% showing significant clogging of the porous structure.

Also, it was found that performing the drying step at 80 °C improves the stability of the PDMS coating on the ME struts. ME/PDMS foams dried at RT for 24 hours loss between 30-50 wt.% of the PDMS coating after 24 hours immersion on ethanol (about 90 wt.% after 10 minutes in THF), while ME/PDMS foams dried at 80 °C for 3 hours present PDMS losses below 10 wt.% related to residues of uncured PDMS (about 30 wt.% after 10 minutes in THF). Moreover, the PDMS base and curing agent weight ratio of 10:1 provides a more stable coating of the ME struts than other ratios proposed in the literature such as 5:1. [1] This ratio, even dried at 80 °C for 3 hours, suffers losses about 30 wt.% when immersed in ethanol (about 80 wt.% after 10 minutes in THF).

Preparation of ME/PDMS/Au and ME/PDMS/Pd foams

As mentioned in the Introduction, the accidental release of NPs to the environment should be avoided. Therefore, after the ME/PDMS/Au and ME/PDMS/Pd foams production, it is necessary to remove any nonstably linked NPs (e.g., NPs not-well attached to the foams or NPs synthesized in the solution and deposited on the foams during the drying process). The remaining water after each

washing cycle was analyzed by UV-Vis spectroscopy, using a Varian Cary 6000i UV-visible-NIR spectrophotometer in double-beam configuration with distilled water as a reference sample. All spectra were recorded in the range from 250 to 800 nm. The presence of NPs was determined by their characteristic plasmon peaks (Figure S2.a). It was found that after 5 washing cycles, no NPs were released. Moreover, after the washing procedure, the obtained foams can be kept in water under shaking for 24 hours without any release (Figure S2.b).

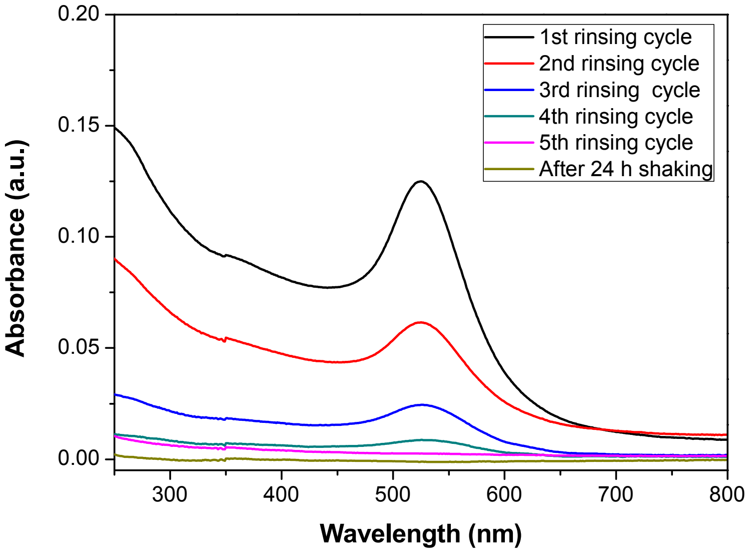

Figure S2. UV-Vis spectra of the remaining water after 1, 2, 3, 4, and 5 rinsing cycles with distilled water of a ME/PDMS/Au foam (about 1 gold wt.%). As observed, after five rinsing cycles, no signal from residues or NPs can be detected (no relevant signals are present over 250 nm). On the contrary, from one to four rinsing cycles, a peak can be observed about 530 nm, which is related to non-attached gold NPs. Also, once the rinsing procedure is completed, the samples can be kept in water under shaking for 24 hours without releasing NPs or any residues.

X-ray Diffraction (XRD) measurements were performed on the ME/PDMS/Au and ME/PDNS/Pd foams using a PANalytical Empyrean X-ray diffractometer equipped with a 1.8 kW CuKα ceramic X-ray tube (l. 1.5418 °A), PIXcel [3D] 2 × 2 mm2 area detector and operating at 45 kV and 40 mA. In both cases, crystalline metallic nanoparticles were identified (Au or Pd, respectively) (Figure S3).

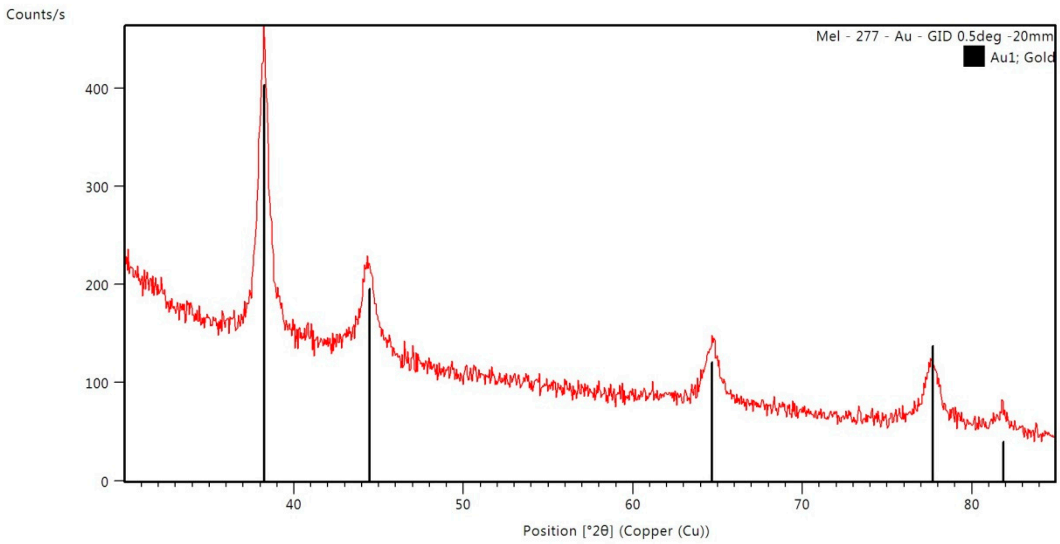

(a)

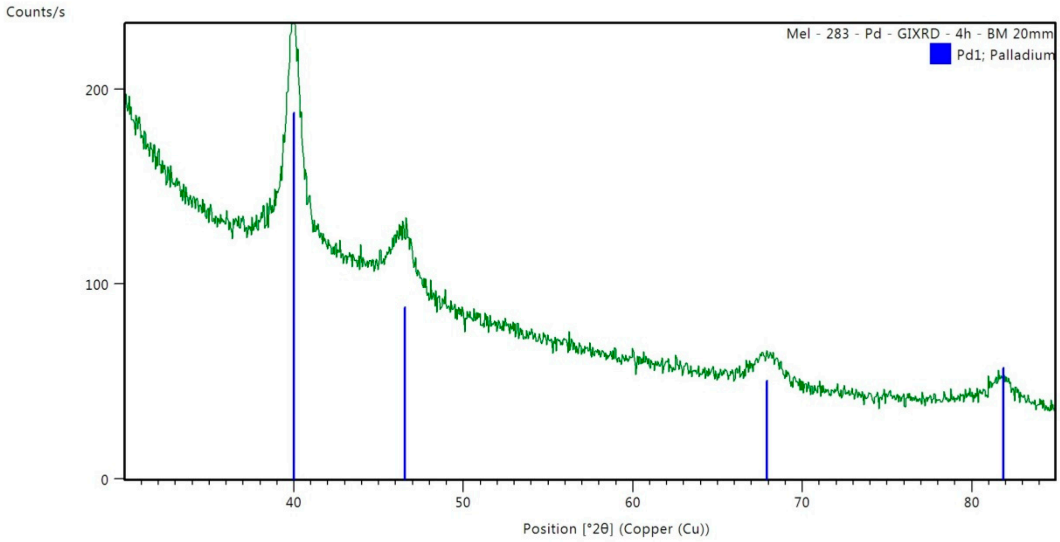

(b)

Figure S3. X-ray diffractograms of the ME/PDMS/Au **(a)** and ME/PDMS/Pd **(b)** foams, showing the characteristic peaks of metallic Au and Pd.

After the ME/PDMS/Au and ME/PDMS/Pd foams production, it was possible to analyze the spatial distribution of the PDMS coating and the NPs by HRSEM. Some struts presented small regions not completely covered by PDMS, being possible to prove that the presence of the PDMS layers is required for the *in-situ* synthesis of the NPs (Figure S4).

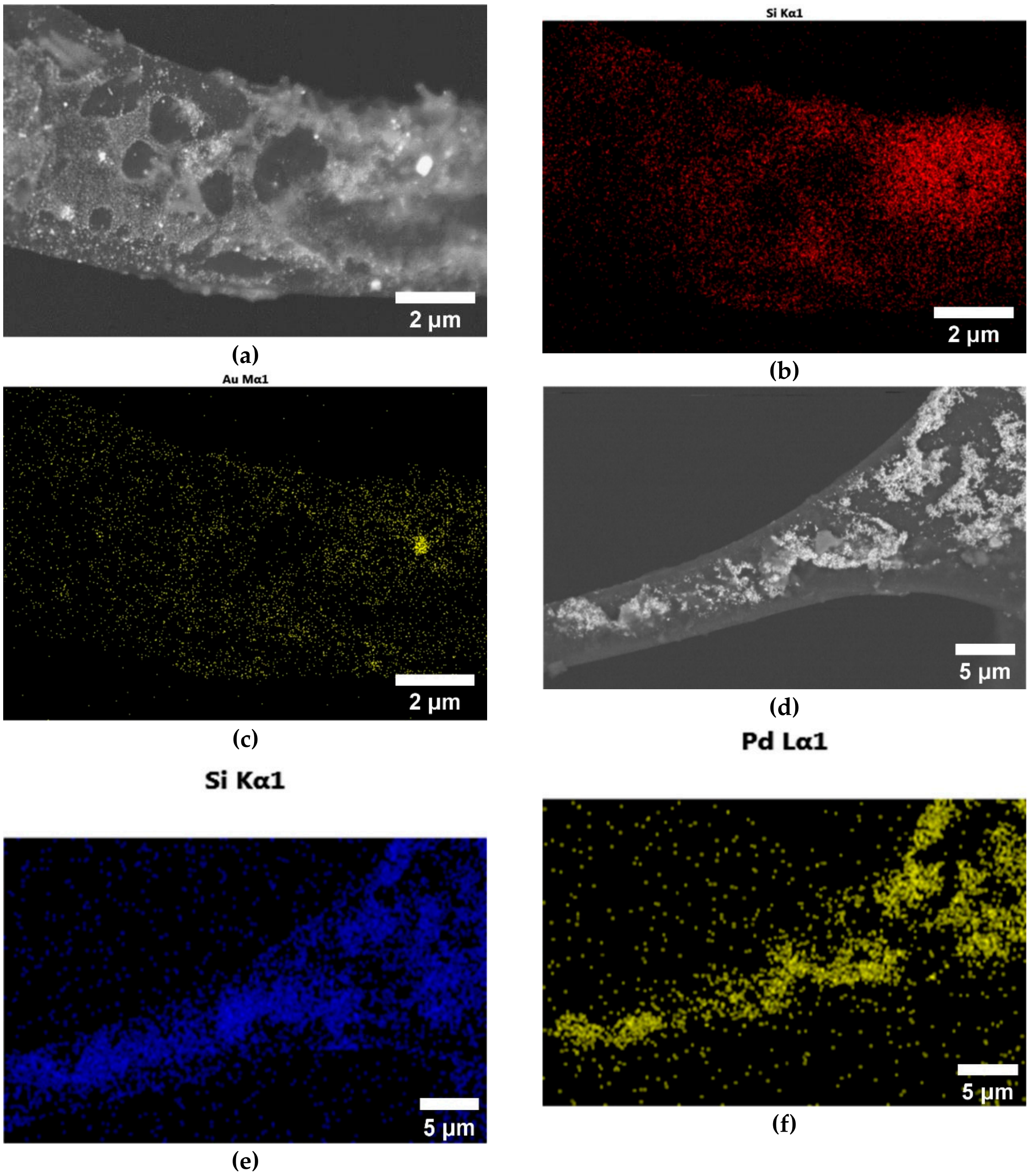

Figure S4. HRSEM micrograph of a ME/PDMS/Au foam obtained after 48 hours in contact with a 0.70 mg/mL solution of $HAuCl_4$ in ethanol **(a)** and the corresponding EDS maps showing the direct relationship between the presence of Si from the PDMS **(b)** and the formation of Au NPs **(c)**. SEM micrograph of a ME/PDMS/Pd foam obtained after 15 hours in contact with a 0.50 mg/mL solution of Na_2PdCl_4 in ethanol **(d)** and the corresponding EDS maps showing the direct relationship between the presence of Si **(e)** from the PDMS and the formation of Pd NPs **(f)**.

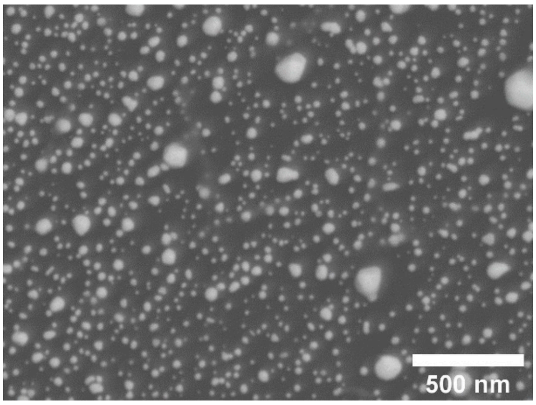

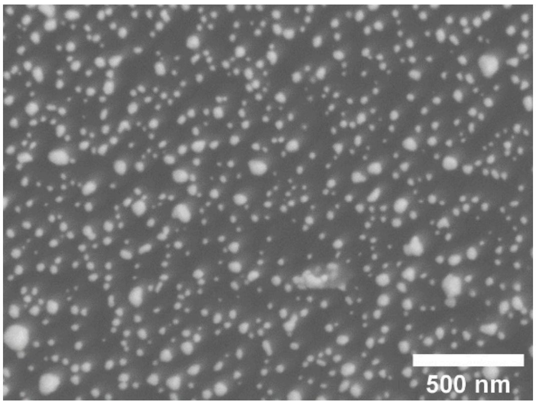

(a) **(b)**

Figure S5. HRSEM micrograph of the struts of a ME/PDMS/Au foam before **(a)** and after **(b)** being kept in water for 24 under shaking, showing no alteration of the presence of NPs on the struts.

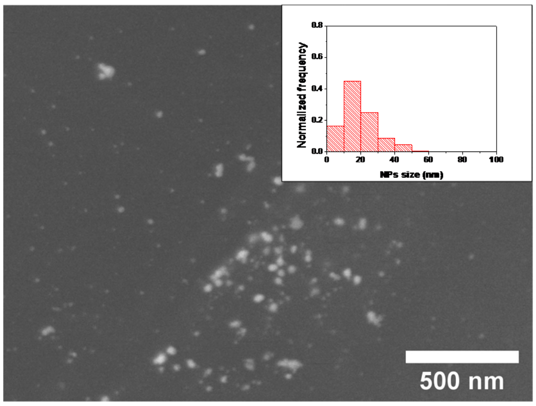

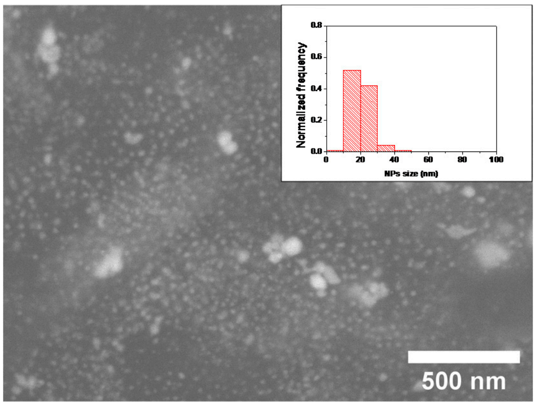

(a) **(b)**

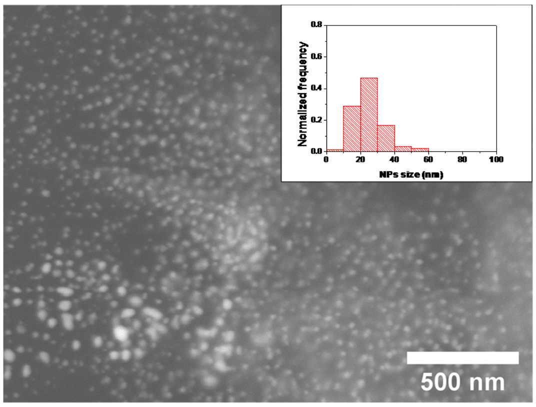

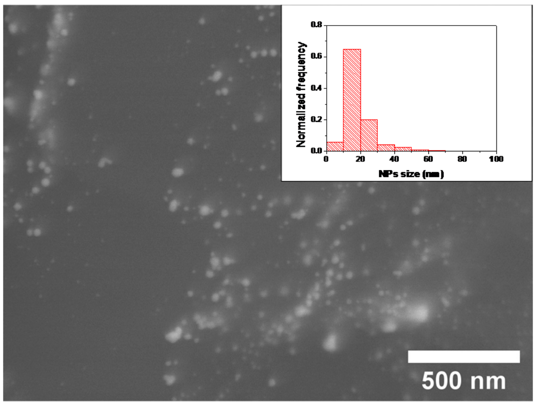

(c) **(d)**

Figure S6. HRSEM micrographs and NPs size distribution (insets) of the ME/PDMS/Au foams obtained after different reaction times in 0.35 mg/mL of $HAuCl_4$ in ethanol: 3 **(a)**, 6 **(b)**, 15 **(c)**, and 48 h **(d).**

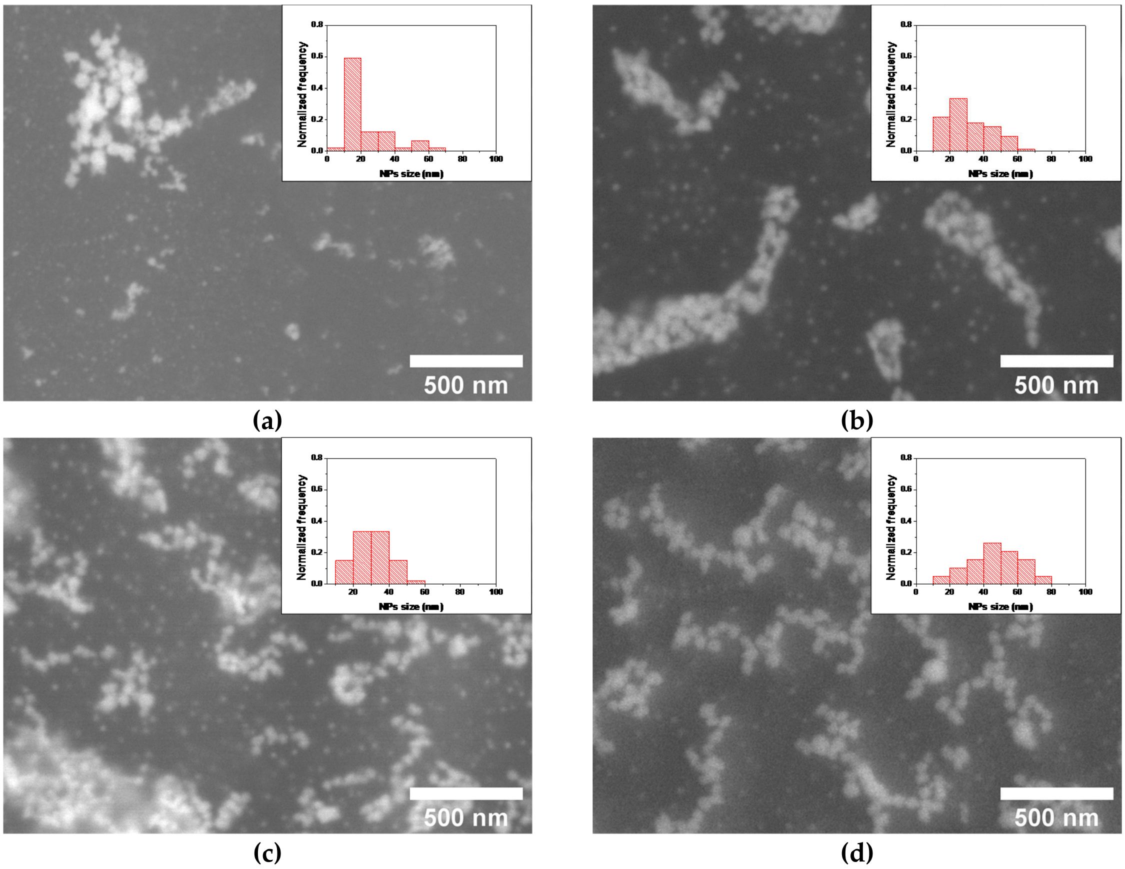

Figure S7. HRSEM micrographs and NPs size distribution (insets) of the obtained ME/PDMS/Pd foams obtained after different reaction times in 0.50 mg/mL of Na_2PdCl_4 in ethanol: 3 **(a)**, 6 **(b)**, 15 **(c)**, and 48 h **(d)**.

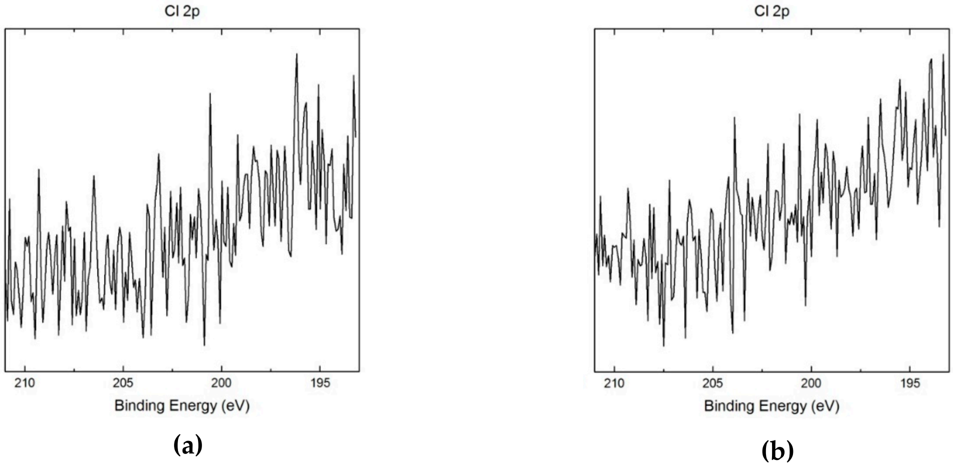

Figure S8. High-resolution XPS spectra of ME/PDMS/Au **(a)** and ME/PDMS/Pd **(b)** centered at binding energies corresponding to Cl 2p showing no remaining of the precursor salts.

Preparation of PU/PDMS/Au and PU/PDMS/Pd foams

The proposed approach can be applied to other polymer foams, such as polyurethane foams. PU/PDMS/Au and PU/PDMS/Pd foams were obtained following the proposed approach. A polyurethane foam, kindly provided by Recticel Flexible Foams Inc., with a density of 30 kg/m^3 and open cell structure with average sizes of about 500 μm, was employed successfully as substrate (Figure S9).

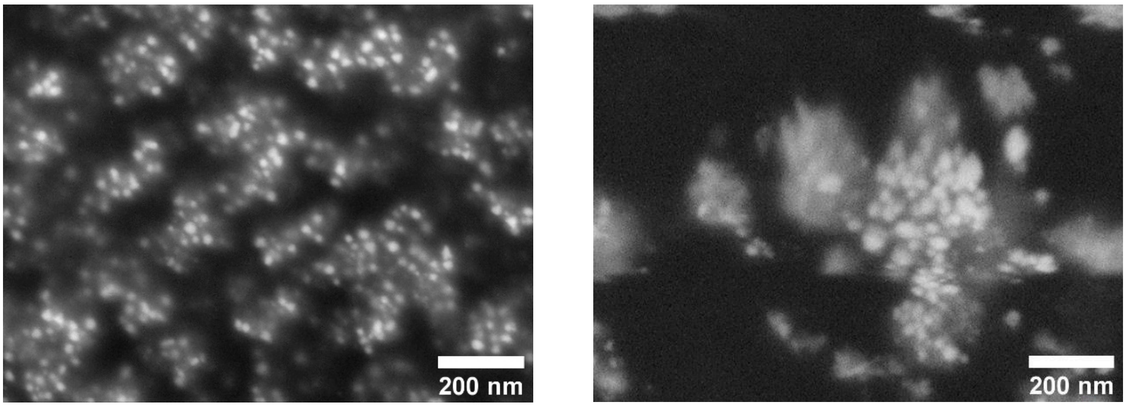

(a) **(b)**

Figure S9. HRSEM micrographs of the surface of the struts of PU/PDMS/Au **(a)** and PU/PDMS/Pd **(b)** foams showing the presence of Au and Pd NPs, respectively.

References:

1. Calcagnile, P.; Fragouli, D.; Mele, E.; Ruffilli, R.; Athanassiou, A. Polymeric foams with functional nanocomposite cells. *RSC Adv.* **2014**, *4*, 19177.
